# *In Silico* Finding of Key Interaction Mediated α3β4 and α7 Nicotinic Acetylcholine Receptor Ligand Selectivity of Quinuclidine-Triazole Chemotype

**DOI:** 10.3390/ijms21176189

**Published:** 2020-08-27

**Authors:** Kuntarat Arunrungvichian, Sumet Chongruchiroj, Jiradanai Sarasamkan, Gerrit Schüürmann, Peter Brust, Opa Vajragupta

**Affiliations:** 1 Department of Pharmaceutical Chemistry, Faculty of Pharmacy, Mahidol University, 447 Sri-Ayutthaya Road, Bangkok 10400, Thailand; kuntarat.aru@mahidol.ac.th; 2 Department of Microbiology, Faculty of Pharmacy, Mahidol University, 447 Sri-Ayutthaya Road, Bangkok 10400, Thailand; sumet.cho@mahidol.ac.th; 3 Division of Nuclear Medicine, Department of Radiology, Faculty of Medicine, Khon Kaen University, 123 Mittraparp Highway, Khon Kaen 4002, Thailand; jirasar@kku.ac.th; 4 UFZ Department of Ecological Chemistry, Helmholtz Centre for Environmental Research, Permoserstrasse 15, 04318 Leipzig, Germany; gerrit.schuurmann@ufz.de; 5 Institute of Organic Chemistry, Technical University Bergakademie Freiberg, Leipziger Strasse 29, 09596 Freiberg, Germany; 6 Department of Neuroradiopharmaceuticals, Institute of Radiopharmaceutical Cancer Research, Helmholtz-Zentrum Dresden-Rossendorf, Permoserstrasse 15, 04318 Leipzig, Germany; p.brust@hzdr.de; 7 Office of Research Affairs, Faculty of Pharmaceutical Sciences, Chulalongkorn University, 254 Phayathai Road, Bangkok 10330, Thailand

**Keywords:** stereoselectivity, anti-1,2,3-triazole, α7 nAChR, α3β4 nAChR, quinuclidine

## Abstract

The selective binding of six (*S*)-quinuclidine-triazoles and their (*R*)-enantiomers to nicotinic acetylcholine receptor (nAChR) subtypes α3β4 and α7, respectively, were analyzed by *in silico* docking to provide the insight into the molecular basis for the observed stereospecific subtype discrimination. Homology modeling followed by molecular docking and molecular dynamics (MD) simulations revealed that unique amino acid residues in the complementary subunits of the nAChR subtypes are involved in subtype-specific selectivity profiles. In the complementary β4-subunit of the α3β4 nAChR binding pocket, non-conserved AspB173 through a salt bridge was found to be the key determinant for the α3β4 selectivity of the quinuclidine-triazole chemotype, explaining the 47–327-fold affinity of the (*S*)-enantiomers as compared to their (*R*)-enantiomer counterparts. Regarding the α7 nAChR subtype, the amino acids promoting a however significantly lower preference for the (*R*)-enantiomers were the conserved TyrA93, TrpA149 and TrpB55 residues. The non-conserved amino acid residue in the complementary subunit of nAChR subtypes appeared to play a significant role for the nAChR subtype-selective binding, particularly at the heteropentameric subtype, whereas the conserved amino acid residues in both principal and complementary subunits are essential for ligand potency and efficacy.

## 1. Introduction

Nicotinic acetylcholine receptors (nAChRs), ligand-gated ion channel receptors (LGICs), can be classified by subunit stoichiometry. The identified neuronal subtypes are at least 12 at present, namely α2-α10 and β2-β4 [1,2]. These receptors being activated by the endogenous neurotransmitter acetylcholine (ACh) play an essential role in the regulation of cation permeation across membranes [3]. The nAChRs are involved in several neuronal dysfunctions related to the cholinergic system. Due to different expression areas of the various subtypes in the brain, compounds acting on each subtype show diverse pharmacological profiles [3,4]. In this context, the α7 and α4β2 subtypes located mainly in the central nervous system (CNS) have been initially exploited as drug targets in the discovery of drugs for neurodegenerative diseases [5,6,7]. For example, compounds acting on α7 and α4β2 nAChRs have been developed as drugs for neuropsychiatric disorders such as Alzheimer′s disease, schizophrenia, Parkinson’s disease, and autism [8,9]. Nowadays, α3β4 nAChR has emerged as an attractive therapeutic target for drug addiction as evidenced by its high expression in specific brain regions associated with addiction and reward systems e.g., medial habenula (MHb), interpeduncular nucleus (IPN), and pineal gland [10]. Therefore, compounds acting on α3β4 nAChRs can be used for the treatment of drug addiction [11,12,13].

**Figure 1:**
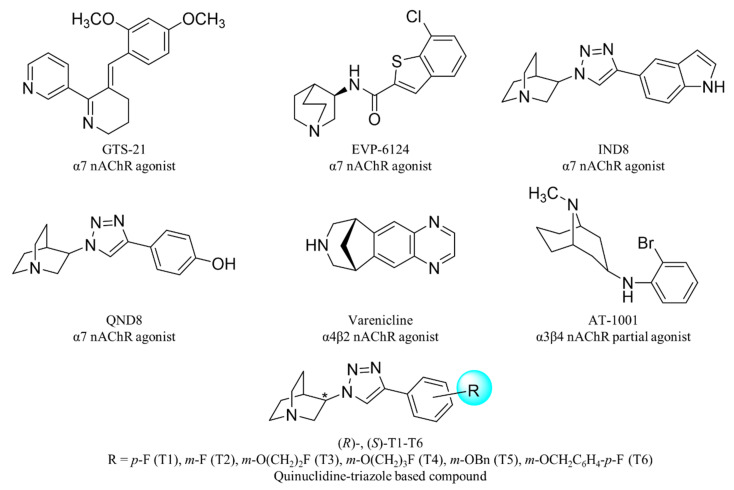
Examples of nicotinic acetylcholine receptor (nAChR) ligands.

In the development of drugs acting on nAChRs, the designs are generally based on ACh pharmacophoric structures, the pharmacophore of nAChR ligands being composed of three key substructures: a cationic center, a hydrogen bond acceptor, and a hydrophobic part [14]. Several compounds in preclinical and clinical studies including GTS-21 [15], EVP-6124 [16,17], varenicline [18,19], and AT-1001 [20,21] (Figure 1) have at least 2 pharmacophore features in common. Recently, we have reported quinuclidine-triazole derivatives as potent nAChR ligands, namely IND8, QND8, and (*R*)-, (*S*)-T1-T6 [22] (Figure 1). These compounds contain an anti-1,2,3-triazole ring in the structure which acts as a linker to connect a hydrophobic part and the remaining pharmacophoric motifs and also serves as the hydrogen bond acceptor. The key observation is that the stereochemistry of quinuclidine at 3-position is an important determinant for subtype-selective binding, particularly the α7 and α3β4 nAChR binding affinities. The (*R*)-enantiomer dominates the binding to the α7 subtype, whereas its (*S*)-counterpart predominantly binds to α3β4 nAChRs [22]. Herein, the computational research into ligand-receptor binding mode which is composed of homology modeling, molecular docking, and a molecular dynamics simulation was performed to identify amino acid residues and interactions mediating the selective binding. This study aimed at a molecular level understanding of the previously observed stereoselective binding of quinuclidine-triazole ligands. The presented rationale for stereoselective nAChR binding may not only provide a guidance for future experimental work but also trigger a future computational research into potent subtype-specific ligands for nAChR especially to α3β4 nAChR ligands for drug addiction treatment.

## 2. Results and Discussion

Our previous study on the nAChR binding affinity of 3-quinuclidine-triazole T1-T6 has revealed that the stereochemistry of quinuclidine at 3-position mediated a subtype-selectivity profile [22]. The information on selective binding of (*R*)-enantiomers to α7 nAChR and (*S*)-enantiomers to α3β4 nAChR is based on the eudismic ratios ((*S*)- vs. (*R*)-enantiomer binding against α3β4 subtype and (*R*)- vs. (*S*)-enantiomer binding against α7 subtype) in Table 1. Herein, the binding modes of these six enantiomeric pairs in protonated form were explored to identify key determinants for α7 and α3β4 nAChR selective binding. The key amino acid determinants provide useful information for the design of a subtype-selective ligand. Hence, homology models of human α7 and α3β4 nAChR were prepared and molecular dockings were performed to investigate the binding modes and to identify the amino acid residues that governed the selectivity. The MD simulation was performed to confirm the determinant residue for subtype selective binding.

### 2.1. nAChR Homology Model Templates (α7 and α3β4 Subtypes)

The homology models of the extracellular domain (ECD) of α7 and α3β4 nAChRs were constructed by Modeller9.15 [23]. The criteria for the template selection are 1) using X-ray diffraction to obtain the crystal structure, 2) having a nAChR agonist as ligand, 3) having a high percentage of similarity in amino acid sequence, and 4) having a high resolution (<2.5 Å). Based on these selection criteria, we have chosen the X-ray crystal structure of acetycholine binding protein (AChBP, a homolog of the nAChR ligand-binding domain) from *Lymnaea stagnalis* (*Ls*-) PDB code 5AFH [24] as the template. Even this template is a chimeric receptor, the carbon backbone is not different from the wild type (PDB code 2BYS) having the same lobeline as a bound ligand. The AChBP has high sequence identity and similarity to the ECD of nAChRs. Its amino acid sequence shows 63.0%, 28.6%, and 28.4% identity to the ECD of human α7, α3, and β4 nAChRs, respectively, whereas the percentage of similarity is higher, that is 71.1%, 53.5%, and 48.2% for human α7, α3, and β4 nAChRs, respectively analyzed by EMBOSS Needle [25]. The AChBP is the most appropriate template to model the ECD of nAChRs because of its considerable secondary structural similarity to the ECD of nAChRs and the high level of sequence identity at the binding site [26]. The key amino acid residues Tyr and Trp in the binding pocket are present in both AChBP and nAChRs. At present, there is only one nAChR crystal structure having both ECD and transmembrane region available in PDB which is the α4β2 nAChR (PDB code 5KXI) [27]. However, this protein structure has been derived from cryo-electron microscopy and the resolution is beyond the limit (3.9 Å). Therefore, the crystal structure of AChBP (PDB code 5AFH) was selected as the protein template in this study. Clustal Omega [28] was used for sequence alignment of the AChBP and the ECD of α7 and α3β4 nAChRs to generate a total of 100 homology models for each of these two receptor subtypes. As the incorrect folding can occur during the alignment process, the quality of the built homology models was evaluated by Modeller energy function and Ramachandran plots in order to select the best models of α7 and α3β4 nAChRs for molecular docking [29]. The Modeller energy functions that were used to assess the quality of the models were the DOPE score and the GA341 score. DOPE score that reveals the stability of protein conformation was ranked at first. The low DOPE score of models indicated that there are small errors of atomic conformational energy between residues and the model has the high tendency to be the native-like model. Another Modeller energy function to evaluate the model quality is the GA341 score. A GA341 score higher than 0.7 indicates a reliable model, defined by ≥ 95% probability of a correct fold. The DOPE and GA341 scores of the best α7 and α3β4 nAChR models among 100 homology models of each subtypes are shown in Table 2, which reveals that the protein conformation of these models are stable and they have the correct folding. The overall structure quality of the best model was subjected to the assessment by Ramachandran plots of backbone dihedral angles (phi (ϕ) and psi (Ψ)) from PROCHECK [30] to view the stereochemical quality of amino acid side chains and highlight regions with unusual geometry. The Ramachandran plots of α7 and α3β4 nAChR homology models are shown in Supplemental Figure S2. There are only few amino acids in disallowed region (0.5% and 0.3% for α7 and α3β4 nAChRs, respectively). The amino acids in this area are far away from the binding pocket and have no effect to the binding affinity of ligands. The results of the quality assessment verify the high quality of the generated homology models so that the models appear appropriate as α7 and α3β4 nAChR templates for molecular modeling.

**Figure 2:**
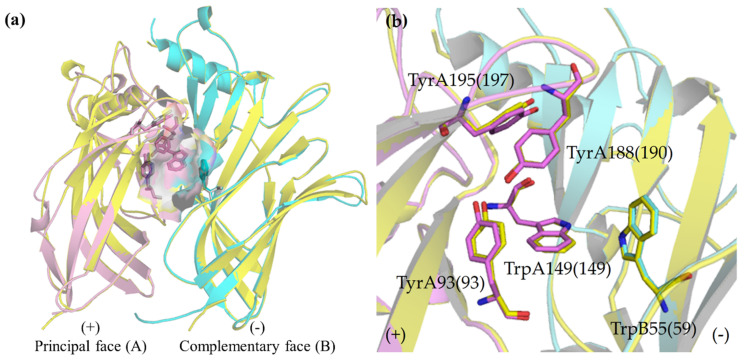
The overlay of (**a**) α7 and α3β4 nAChR quaternary structures with molecular surface of amino acid residues in an aromatic cage and (**b**) amino acid residues in an aromatic cage. The amino acid residues are labeled for the α7 nAChR, whereas the sequence number of residues in the α3β4 nAChR are in a parenthesis. Yellow, α7 nAChR; pink, α3 nAChR; cyan, β4 nAChR; red, oxygen; blue, nitrogen.

To confirm the validity and reliability of the generated homology α7 and α3β4 nAChR models, firstly, the homology models of α7 and α3β4 nAChRs were superimposed to examine the key amino acid residues in the binding sites of the α7 and α3β4 subtypes (Figure 2). The overlay of nAChR quaternary structures indicated that all key or conserved residues in an aromatic cage i.e., TyrA93, TrpA149, TyrA188, TyrA195, and TrpB55 (Supplemental Figure S3) [31,32] aligned in the same configuration as shown in Figure 2a,b represent principal and complementary subunits, respectively; the sequence number of amino acid residues were assigned according to the α7 nAChR. The mentioned amino acid residues are important for ligand binding to form the cation-π, hydrogen bond, and hydrophobic interactions with nAChR ligands [14]. The critical interaction to modulate nAChR affinity and functionality covers cation-π and hydrogen bond interactions between the basic amine of the ligand and the conserved Trp or Tyr residues in the principal subunit [33,34]; these findings support the notion that the selectivity profile of the ligands is likely to be mediated through non-conserved amino acid residues.

**Figure 3:**
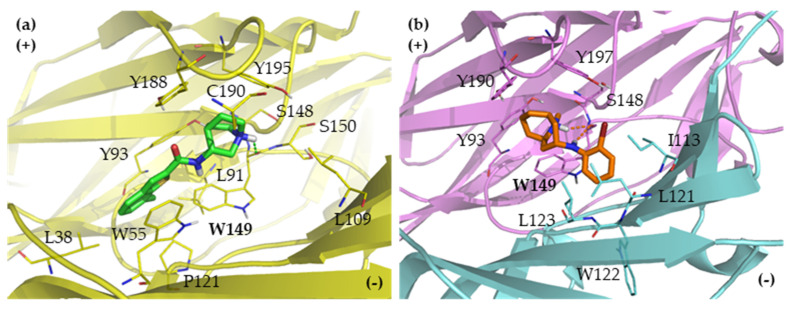
The docked poses of (**a**) EVP-6124 (green), an α7-selective nAChR ligand in the human α7 nAChR homology model (yellow) and (**b**) AT-1001 (orange), an α3β4-selective nAChR ligand in the human α3β4 nAChR homology model (pink and cyan). The bold letters show amino acids forming hydrogen bond interactions. red, oxygen; blue, nitrogen.

Secondly, the nAChR subtype-selective ligands, EVP-6124 [16,17] and AT-1001 [20,21] were docked to the homology models. The docked poses of the nAChR subtype-selective ligands, which are the α7-selective EVP-6124 and the α3β4-selective AT-1001, against the homology models were analyzed to confirm the validity of the prepared homology models (Figure 3, Supplemental Figures S4 and S5). The docked poses of the α7-selective EVP-6124 against the α7 nAChR are displayed in Figure 3a. The quinuclidine ring of EVP-6124 has cation-π interactions (HN^+^---π) with TrpA149 and TyrA195 and hydrogen bond interaction (^+^NH---:O=C carbonyl backbone) with TrpA149, the key amino acid residue in the aromatic cage (Figure 3a, Supplemental Figure S4a). In contrary, the EVP-6124 in the AChBP template and the human α3β4 nAChR homology model flipped horizontally, turning the quinuclidine ring in opposite direction, unable to form either hydrogen bond or cation-π interactions with TrpA149. For the docked poses of the α3β4-selective AT-1001 against α3β4 nAChR, there are the cation-π interactions between the bicyclic granatane motif of AT-1001 and TrpA149 and TyrA197. The H atom of protonated granatane motif has a hydrogen bond interaction with the carbonyl backbone of TrpA149, a key residue (Figure 3b, Supplemental Figure S5). These interactions have not been observed in AChBP, but they have been found in α7 nAChR model because the interacting amino acid residues are conserved amino acids. However, the numbers of clusters when docking in α7 nAChR are more than α3β4 nAChR. The results demonstrate that the nAChR ligands are able to form hydrogen bond and cation-π interactions with several amino acid residues; however, the homology models of the nAChR subtypes (α7 or α3β4 nAChRs) could better accommodate their corresponding selective ligands and provide interactions with the key conserved amino acid residues. These results confirm the validity of using the constructed α7 and α3β4 nAChR homology models for molecular docking studies of quinuclidine derivatives.

The six enantiomeric pairs of stereospecific compounds, the (*R*)- and (*S*)-enantiomers of T1–T6, were then docked with the constructed α7 and α3β4 nAChR homology models by using AutoDock4.2 [35] to investigate the binding modes and to identify the amino acid residues that governed the selectivity. T1–T6 with pK_a_ values of 8.93–8.95 [22] were docked in protonated form that is prevalent to at least 97% at physiological pH. These six quinuclidine-triazole enantiomeric pairs can be divided into two classes concerning the substituent R (Table 1), i.e., the small substituent pairs (T1–T2) and large substituent pairs including long alkyl side chains and large hydrophobic groups (T3–T6).

However, docking-derived free energies of binding have a limited accuracy that is about ±2 kcal/mol for AutoDock [36], preventing a discrimination between (*R*)- and (*S*)-enantiomers with ΔG values typically within this range (at 37 °C, ΔG values of 2 and 4 kcal/mol yield Boltzmann-derived eudismic factors of 26 and 661, respectively). Indeed, the differences in computed free energies of binding between the (*R*)- and (*S*)-enantiomers (Supplemental Table S1) show no significant correlation with the experimentally observed variation in nAChR binding affinity. Accordingly, our focus was on identifying potentially relevant interaction modes (hydrogen bond, cation-π, π-π, electrostatic salt bridge, and hydrophobic interactions), and how these might depend on the stereochemistry of the ligands. In this way, the purpose of the computational docking was to unravel amino acid determinants and interactions responsible for the observed subtype selectivity.

Generally, the docked poses of the largest conformational cluster (the most populated cluster with the highest % member) were visually analyzed for key interaction motifs between ligands and amino acid residues in the binding site of nAChRs. In addition to the largest cluster, the second largest cluster and the cluster with the best ΔG score were also taken into account. The binding poses of the ligands obtained from molecular docking were related to the in vitro α3β4 and α7 nAChR binding affinities reported earlier (Table 1) [22].

### 2.2. Binding Modes

The eudismic ratio calculated from in vitro K_i_ values revealed high stereoselective binding. The (*S*)-enantiomers have 47- to 327-fold higher affinity toward the α3β4 nAChR than their (*R*)-counterparts. Rather high ratios of 160–327 were found for the compounds T1-T2 containing a small substituent on the benzene ring (Table 1). Lower eudismic ratios for the α7 nAChR (1.6- to 12-fold) were observed for the (*R*)-enantiomers. With regard to the subtype selectivity ratios of the single enantiomers between the α3β4 subtype and the α7 subtype, the (*S*)-enantiomers were found to be selective for the α3β4 subtype (5- to 294-fold) while the (*R*)-enantiomers were selective for the α7 subtype to a lower extent (3.1- to 33-fold). The more pronounced stereoselectivity of the (*S*)-enantiomers toward the α3β4 nAChR than that of the (*R*)-enantiomers toward the α7 nAChR is likely due to the different subunits comprising the aromatic cage, particularly the complementary β4 chain, which is replaced by another α7 subunit in the α7 nAChR. Hence, the molecular dockings of six enantiomeric pairs were performed and the obtained binding modes were extensively investigated to give structural insights ( Supplemental Figures S6–S9) into the stereoselective binding of quinuclidine-triazole compounds as detailed below.

#### 2.2.1. Binding Modes of (*S*)-Enantiomers

**Figure 4:**
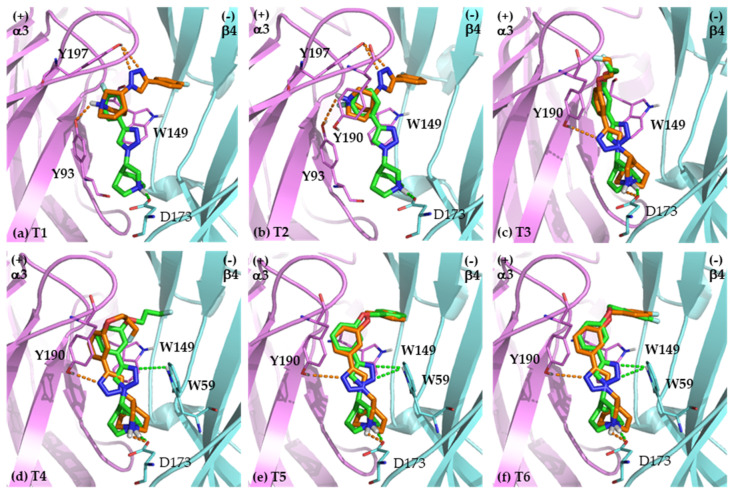
The docking poses to α3β4 nAChR of the (*R*)-enantiomers (orange) and the (*S*)-enantiomers (green) of T1-T6 (**a**–**f**). The amino acid residues forming hydrogen bond, cation-π, and π-π interactions are shown. The polar hydrogen atoms of the interacting amino acid side chains are displayed to identify hydrogen bond donors and acceptors. The conserved residues are shown in bold. Pink, α3 nAChR; cyan, β4 nAChR; red, oxygen; blue, nitrogen; orange dashed lines, hydrogen bond interaction with (*R*)-enantiomers; green dashed lines, hydrogen bond interaction with (*S*)-enantiomers.

The docked ligands or poses in major clusters have been analyzed. All (*S*)-enantiomers aligned nicely in the binding pocket of α3β4 nAChRs, which is an aromatic cage located at the interface between the α3 subunit (chain A, principal side) and the β4 subunit (chain B, complementary side). For the binding modes of compounds T1-T2 bearing small substituents (*R*) on the benzene ring, the main components (quinuclidine, triazole, and benzene ring) of (*R*)- and (*S*)-enantiomers aligned in different orientation (Figure 4a,b). Whereas compounds T3-T6 contain long aliphatic and aromatic R, particularly T5 and T6 (Figure 4c–f), the alignments of these motifs in each enantiomeric pair are in proximity. Comparing the sizes of aromatic cage and the ligands, the small T1 and T2 are able to move freely in the aromatic cage and thus may undergo more stabilizing interactions. T1 and T2 have only one cluster (100% of members), while compounds with substituents showing conformational flexibility (T3–T4) have higher numbers of clusters than less flexible ligands (T1–T2, Supplemental Table S1). The docked poses for the compounds containing large substituents (T5–T6) are more similar to their corresponding counterparts than the smaller T1–T2. It is due to the steric hindrance of the additional benzene ring that restricts the ability to conform freely in the binding pocket.

The (*S*)-enantiomers are able to form hydrogen bond or cation-π and π-π interactions with the conserved amino acid residues in both α3-principal and β4-complementary sides of the aromatic cage (Table 3). The interactions between (*S*)-enantiomers and the amino acid residues in both subunits (α3 and β4) indeed stabilize and strengthen the binding resulting in the low affinity values (K_i_ = 2–19 nM, Table 1). Although the (*R*)-enantiomers of compounds T3–T6 were able to interact with AspB173 in the β4 complementary subunit, the % member in the largest clusters and the length of hydrogen bond interactions in the binding modes of compounds (*R*)-T3 to (*R*)-T6 were respectively lower and longer than those of the corresponding (*S*)-enantiomers. The orders of magnitude of the in vitro binding affinities appear to be different between the various compounds. It is possibly due to distinct binding poses of each enantiomeric pair (Figure 4). In term of the interacting residues in each subunit (principal subunit compared to the complementary subunit), the (*S*)-enantiomers with higher affinity tend to form hydrogen bonds with amino acids in the complementary subunit than the (*R*)-enantiomers (Table 3). The shorter hydrogen bonds (1.65–1.78 Å for (*S*)-enantiomers vs. 1.95–2.08 Å for (*R*)-enantiomers) correspond to larger bond strengths, and thus translate into higher ligand-receptor affinities. These findings provide a mechanistic rationale for the experimental outcome that the α3β4 subtype selectivities of the (*S*)-enantiomers are 5 to 294 times higher than those of their enantiomeric counterparts (Table 1). Accordingly, the present molecular docking results strongly suggest that the selective α3β4 binding of the (*S*)-enantiomers at the C-3 of quinuclidine in quinuclidine-triazole ligands is mediated by interactions with amino acid residues in the complementary face.

#### 2.2.2. Binding Modes of (R)-Enantiomers

**Figure 5:**
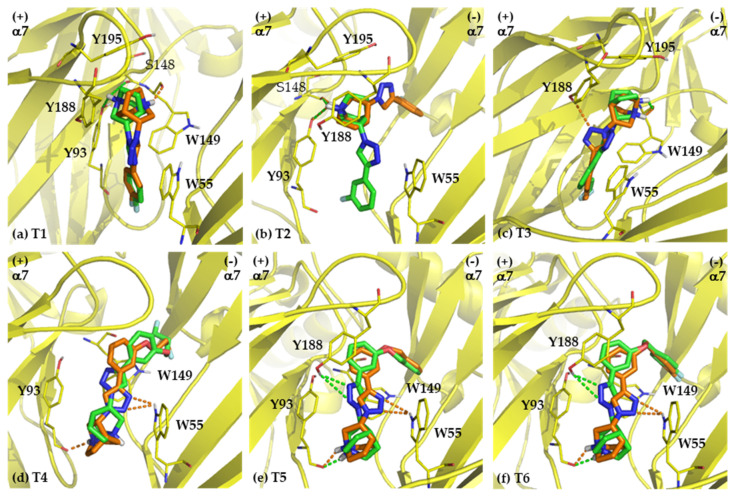
The docking poses to the α7 nAChR of the (*R*)-enantiomers (orange) and the (*S*)-enantiomers (green) of T1-T6 (**a**–**f**). The amino acid residues forming hydrogen bond, cation-π, and π-π interactions are shown. The polar hydrogen atoms of the interacting amino acid side chains are displayed to identify hydrogen bond donors and acceptors. The conserved residues are shown in bold. Yellow, α7 nAChR; red, oxygen; blue, nitrogen; orange dashed lines, hydrogen bond interaction with (*R*)-enantiomers; green dashed lines, hydrogen bond interaction with (*S*)-enantiomers.

The binding poses of all enantiomeric pairs located in the binding site of the α7 nAChR and the orientations are nearly the same except for compound T2 (Figure 5). Although the triazole ring and the hydrophobic part of all the other enantiomeric pairs are almost superimposable, the quinuclidine ring, particularly the N atom appeared to shift slightly from its counterpart. This might be caused by the rigidity of the triazole linker and the phenyl ring of the hydrophobic part that limit the rotation and conformational freedom of the pose in the binding pocket. Visual analysis of the binding interactions revealed that all enantiomeric pairs are aligned within the aromatic cage and mostly formed interactions with the conserved amino acid residues of the nAChR composed of TyrA93, TrpA149, TyrA188, and TyrA195 in the principal subunit, and of TrpB55 in the complementary subunit (Table 3). These interactions of the (*R*)-enantiomers lead to substantial binding to the α7 nAChR (K_i_ = 22–117 nM) but moderate selectivity. The subtype selectivity ratios are 3.1–33 compared to the 5–294 selectivity ratios of the (*S*)-enantiomers toward the α3β4 nAChR (Table 1). The α7 nAChR subtype selectivity is low for the (*R*)-enantiomers because the interacting residues are the conserved amino acids, available in all nAChR subtypes. The eudismic ratios of the (*R*)-enantiomers over the (*S*)-enantiomers toward the α7 nAChR are considered to be moderate as well (1.6- to 12-fold) because the docked poses of each enantiomeric pair share a large common part in the same alignment (triazole ring and hydrophobic part) and thus provide similar binding interactions with the residues in the principal subunit (Figure 5). The only distinct interaction between the (*R*)- and the (*S*)-enantiomers, which has little effect on the α7 nAChR binding affinity, is related to hydrogen bond interactions between the triazole ring and a conserved amino acid residue, TrpB55, in the complementary subunit for (*R*)-enantiomers and TyrA188 in the principal subunit for (*S*)-enantiomers.

### 2.3. Identification of Amino Acid Determinants and Interactions for the Stereoselective Binding

The different alignment of the binding poses to the α3β4 nAChR between the highly selective (*S*)-quinuclidine-triazole compounds (47- to 327-fold selective) and its (*R*)-counterparts as well as the more favorable binding of single (*S*)-enantiomers to the α3β4 nAChR over the α7 nAChR (5- to 294-fold more selective) inspired further exploration to identify amino acid residues which are responsible for the selectivity. To identify crucial amino acid residues and interactions, the interacting residues and interactions in the binding modes obtained from molecular docking were visually analyzed.

#### 2.3.1. Determinants of Selective α3β4 Binding

In the comparison of interacting amino acid residues between each (*R*)- and (*S*)-enantiomer against the α3β4 nAChR, the (*S*)-enantiomers provided interactions to both α3 and β4 subunits (chain A and chain B), whereas the (*R*)-enantiomers predominately interacted with the principal α3 subunit. In fact, the small molecules, (*R*)-T1 and (*R*)-T2 bind only to the α3 principal subunit. The most common interacting residues of the (*S*)-enantiomers are the conserved residues, TrpA149 (π-π) and TyrA190 (π-π) in the principal α3 subunit, and the conserved TrpB59 (hydrogen bond) and the non-conserved AspB173 (hydrogen bond) in the complementary β4 subunit (Table 3). In all (*S*)-enantiomers, the protonated H-atom of N-quinuclidine forms hydrogen bond with an O-atom in the carboxylate side chain of AspB173 within a range of 1.65–1.78 Å (Table 3). Notably, (*S*)-T1 and (*S*)-T2 exhibited higher affinity and selectivity at the α3β4 nAChR than other (*S*)-enantiomers in the series in spite of less number of interactions. By contrast, the (*R*)-enantiomers showed a respective weaker hydrogen bond (1.95–2.08 Å) only for (*R*)-T3-T6. In addition, % member of docked poses in the largest cluster of (*S*)-T3 to (*S*)-T6 were more than those of (*R*)-T3 to (*R*)-T6, 60–95% vs. 42–83% (Supplemental Table S1). Accordingly, (*S*)-T3 to (*S*)-T6 can better conform to this binding mode that may contribute to the selective binding of (*S*)-T3 to (*S*)-T6 toward α3β4 nAChR. Therefore, the observed 47- to 327-fold affinity ratio of the (*S*)-enantiomers to the α3β4 nAChR over their (*R*)-enantiomer counterparts is related to (i) stronger interactions with the complementary β4 subunit than with the principal α3 subunit, (ii) the difference in capability for stabilizing interactions with the crucial AspB173, and (iii) % member in the most populated cluster corresponding to higher fractions of affinity-maximizing conformations. Based on the remarkably high affinity and selectivity of the (*S*)-enantiomers toward the α3β4 subtype, it is plausible that the AspB173 determinant might confer an electrostatic salt bridge interaction between quinuclidine N^+^ and the ionized AspB173 accompanying the hydrogen bond (^+^NH and lone pair electron of one of the two carboxylate groups of AspB173). The salt bridge (Coulomb electrostatic attraction) is stronger than hydrogen bond and cation-π interactions; however, salt bridges are not visualized by AutoDock and Molecular Operating Environment (MOE) used in this study. Taken together, the high affinities to the α3β4 nAChR result from the ability to form electrostatic and hydrogen bond interactions with Asp173. Moreover, the eudismic ratios regarding α3β4 apparently relate also to the higher numbers of suitable (*S*)-enantiomer conformations in the largest cluster (as expressed by their % members). For instance, (*S*)-T1 and (*S*)-T2 with high eudismic ratios have 100% member in the largest cluster, whereas the low eudismic ratio of (*S*)-T3 is accompanied by a significantly lower population of 60% of the largest cluster. The AspB173 position of the α3β4 subtype is occupied by the homologous GlyB67 residue of the complimentary α7 subtype. However, GlyB167 has no side chain for a correspondingly stabilizing interaction with the ligand. Hence, the AspB173 residue in the complementary face (β4) is considered to be a key determinant residue for the α3β4-selective binding. In addition to AspB173, the amino acid residue that might also play a role in α3β4 subtype selectivity is TrpB59. The TrpB59, however, is a conserved amino acid of nAChRs and can be found in other nAChR subtypes as well, e.g., TrpB55 in the α7 nAChR. Therefore, the role of conserved Trp in complementary subunit could be responsible for affinity and efficacy rather than selectivity.

Moreover, it is interesting to see whether the AspB173 residue in the complementary face (β4) is also a determinant residue for an α3β4-selective binding to other structural scaffolds or chemotypes. To this end, we investigated the binding mode of AT-1001 as selective α3β4 nAChR ligand featuring a different chemotype (Supplemental Figure S5). The hydrogen bond with AspB173 was not observed in the binding mode of AT-1001. Instead, AT-1001 provided more hydrophobic interactions with other residues in the β4 subunit i.e., the non-conserved residues: IleB113, LeuB121, TrpB122, and LeuB123 (Supplemental Figure S5b). AspB173 is therefore not a determinant residue for α3β4-subtype selectivity concerning other chemotypes. These findings suggest that the α3β4-subtype selectivity is governed by interactions with residues in the β4 subunit, particularly regarding the non-conserved residues. This is in agreement with previous studies showing that non-conserved amino acid residues particularly in the complementary subunit have an important role to mediate the binding affinity. For example, Harpsoe et al. [37] studied the roles of the β2 and β4 complementary subunits for cytosine and NS3861 binding. They reported that the interactions with the α subunits are important for agonist efficacy, but the weaker interactions with the β complementary subunits are responsible for fine tuning of the agonist efficacy. The dominant role of the complementary subunit for the binding affinity was also reported by Parker et al. [38]. Ligands such as epibatidine, acetylcholine, nicotine, and dimethylphenylpiperazinium show higher affinities to the β2 than the β4 subunit when having α2 as principal subunit. In fact, key determinants for the α3β4 selective binding have been reported but only for peptide analogs of α-conotoxin (RegIIA and mutated LsIA) [39]. Lys61 and Arg113 were the key determinants for RegIIA [40], while Lys57, Ile177, and Ile109 were the key residues for ligand binding in mutated LsIA [41]. The reported determinants are non-conserved amino acid residues in the complementary subunit and are unique for particular chemotypes.

**Figure 6:**
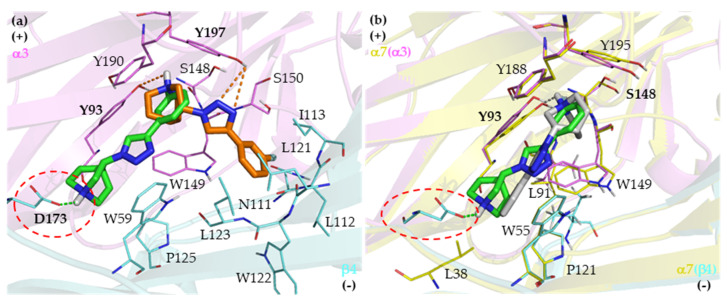
(**a**) The docking poses of (*R*)-T2 (orange) and (*S*)-T2 (green) showing the interacting residues with the α3β4 nAChR and (**b**) the overlay between the (*S*)-T2 docking pose in the α3β4 (green) and the α7 nAChR (grey). The amino acid residues in Figure 6a represent the α3β4 homology model, whereas the amino acid residues in Figure 6b represent the α7 nAChR subunit. The bold letters show amino acids forming hydrogen bond interactions. The polar hydrogen atoms of the amino acid side chains are displayed to identify hydrogen bond donors and acceptors. Pink, α3 nAChR; cyan, β4 nAChR; yellow, α7 nAChR; red, oxygen; blue, nitrogen.

The representative binding modes of compound T2 are displayed in Figure 6. The binding poses of (*S*)-T2 and (*R*)-T2 for the α3β4 nAChR in Figure 6a appeared to be different, both in the alignment (Figure 6a) and the binding interactions (Table 3). (*S*)-T2 was able to form hydrogen bond interactions with amino acid residues in the β4-complementary subunit that are AspB173 (1.77 Å) resulting in the 161-fold higher affinity of (*S*)-T2 to α3β4 nAChR, whereas (*R*)-T2 was able to form hydrogen bond interactions with amino acid residues in the α3-principal subunit (TyrA93 and TyrA197). (*S*)-T2 interacted with residues in both subunits of α3β4 subtype but it mostly interacted with residues in the principal subunit of the α7 subtype only (Figure 6b). These are plausible reasons why (*S*)-T2 was 294-fold more active to the α3β4 subtype than to the α7 subtype.

**Figure 7:**
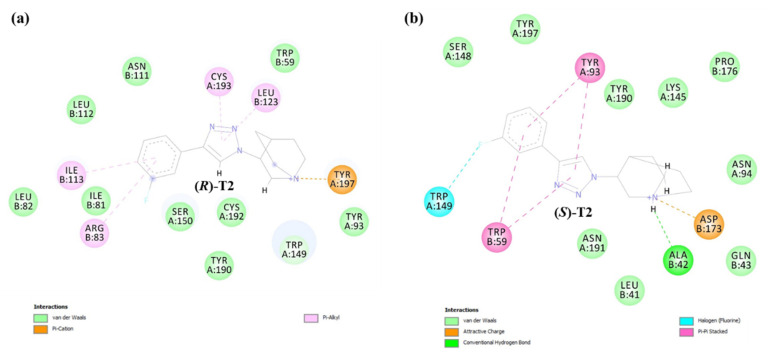
Molecular dynamics (MD) simulation of (**a**) (*R*)-T2 and (**b**) (*S*)-T2 with the α3β4 nAChR.

To gain insight into the AspB173 determinant engaged in salt bridge formation and to explain the highest binding affinity of compound (*S*)-T2 toward α3β4 nAChR subtype (K_i_ = 2.25 nM), MD simulation of T2, both (*S*)- and (*R*)-enantiomers with α3β4 homology model was performed. MD simulation is able to detect the salt bridge formation between the carboxylate of AspB173 determinant and the protonated nitrogen atom of (*S*)-T2 (Figure 7b), which supports our earlier speculation. When compared with molecular docking, the interacting amino acid residues from MD simulation were mostly the same with some discrepancies in types of interaction due to the flexible and solvated receptor in MD simulation. The conformational flexibility of receptor apparently strengthens the binding affinity by allowing α3β4 nAChR subtype to accommodate and stabilize (*S*)-T2 into the binding site. The key interactions of (*S*)-T2 in the α3β4 nAChR binding site are one salt bridge to AspB173, four π-π stacking interactions (TrpB59 and TyrA93), one hydrogen bond interaction with AlaB42 and one halogen bond (TrpA149) which are more than those of (*R*)-T2. The binding mode of (*S*)-T2 with more interactions both in strength and number explains why (*S*)-T2 exhibited the highest binding affinity to α3β4 nAChR subtype.

For compounds T3–T6 with larger substituents than the fluorine atom on the benzene ring, more interactions were found between the (*S*)-enantiomers and the residues in the complementary β4 subunit (non-conserved AspB173 and the conserved TrpB59). Therefore, the key amino acid residues mediating the α3β4-selectivity of the quinuclidine-triazole compounds with substituents larger than the fluorine atom are TrpB59 and AspB173. Experimentally mutated amino acid residues for TrpB59 and AspB173 would help to substantiate the determinant role of these residues for α3β4-subtype selectivity.

#### 2.3.2. Determinants of Selective α7 Binding

The selectivity of the (*R*)-enantiomers for the α7 subtype was not as high as that of the (*S*)-enantiomers for the α3β4 subtype. The noticeable enantiomeric selectivity ratios of compounds T2, T5, and T6 to the α7 nAChR (4- to 12-fold more selective) were found to be associated with the binding interactions, predominately with the conserved residues in the principal and complementary subunits. The crucial interactions are likely to be hydrogen bonds with TyrA93 and TrpB55 (Table 3). The interactions via these amino acid residues except TrpB55 are also found in the binding modes of the (*S*)-enantiomers; therefore, the interacting amino acid residue that mediates the distinct α7 nAChR affinity of this compound series is likely to be TrpB55. Despite the amino acids triggering the α7 nAChR selectivity, we have also found out that the interactions driving the α7 nAChR affinity of quinuclidine-triazole series might be related to hydrophobic rather than cation-π or hydrogen bond interactions as observed in compounds T5-T6 with substituents R larger than the fluorine atom. The additional hydrophobic and π-π interactions of triazole and benzene rings with TrpA149 in (*R*)-enantiomers of compounds T5-T6 (Table 3) led to higher affinity to the α7 nAChR (K_i_ = 22.5–33.2 nM) than (*R*)-T1 and T2 (K_i_ = 73–117 nM) which have only a small fluoride as substituent.

**Figure 8:**
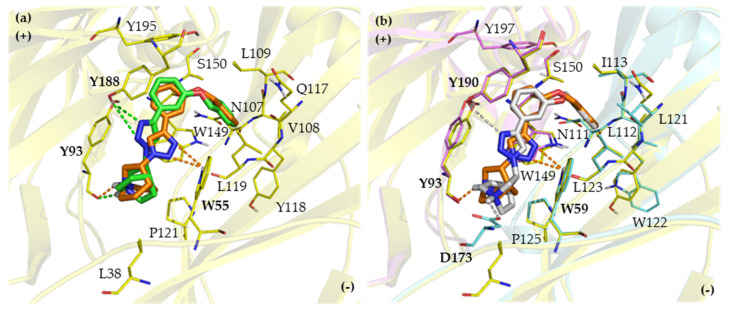
(**a**) The overlay docking poses of (*R*)-T5 (orange) and (*S*)-T5 (green) in the binding site of the α7 nAChR and (**b**) the overlay of (*R*)-T5 docking poses in the α7 (orange) and the α3β4 nAChR (grey).The amino acid residues in Figure 8a represent the α7 homology model, whereas the amino acid residues in Figure 8b belong to the α3β4 nAChR subunit. The bold letters show amino acids having hydrogen bond interactions. The polar hydrogen atoms of the amino acid side chains are displayed to identify hydrogen bond donors and acceptors. Yellow, α7 subunit; pink, α3 subunit; cyan, β4 subunit; red, oxygen; blue, nitrogen.

The representative binding modes of T5 enantiomers are displayed in Figure 8. The binding poses of the (*R*)- and (*S*)-enantiomers for the α7 nAChR depicted in Figure 8a are almost in the same orientations, only triazole ring tilts are found which turn the triazole ring to form hydrogen bond interactions with different amino acid residues. This alignment leads to the ability of (*R*)-T5 to form hydrogen bond interactions with TrpB55 (3.01 and 3.09 Å) and TyrA93 (1.94 Å) as shown in Figure 8a, which can be observed only in (*R*)-T5 and (*R*)-T6 leading to 12-fold and 4.5-fold higher affinity than their (*S*)-counterparts, respectively. The additional π-π interaction between the triazole rings and the benzene ring of TrpA149 also helps to stabilize the α7 nAChR affinity (Figure S8). This selectivity profile of the (*R*)-enantiomers toward the α7 subtype was not as high as observed for the (*S*)-enantiomers toward the α3β4 nAChR because most of the binding interactions of (*R*)-T5 are related to interactions with conserved amino acid residues. The binding motifs which included hydrogen bond interactions with TyrA93 and TrpB55 together with the additional hydrophobic interactions with TrpA149, boosted (*R*)-T5 up to be the most potent α7 nAChR ligand of the series with high selectivity for this subtype.

## 3. Materials and Methods

### 3.1. Protein Template and Ligand Preparations

The amino acid sequences of human α7 and α3β4 nAChRs were downloaded from UniProt for searching a proper protein template from Protein Data Bank (PDB) by Blast protein in Chimera 1.10.2 (Resource for Biocomputing, Visualization, and Informatics at the University of California, San Francisco, CA, USA) [42]. The exclusion criteria was proteins having an antagonist as a ligand. The sequence of the target protein and the template that is the X-ray crystal structure of acetylcholine binding protein (AChBP) in complex with lobeline (PDB ID 5AFH) was aligned by Clustal Omega (Conway Institute, University College Dublin, Dublin, Ireland) [28] and the homology model was generated by Modeller9.15 (University of California San Francisco, San Francisco, CA, USA) [23]. The model quality was evaluated by several parameters i.e., the Discrete Optimized Protein Energy (DOPE) score [43], the GA341 score [29], and the Ramachandran plot [44]. The DOPE score was ranked first. Then, the model was finally selected based on the GA341 score and values from the Ramachandran plot and used as the protein template to study the binding modes of nAChR ligands via molecular docking using AutoDock4.2 program suit (The Scripps Research Institute, San Diego, CA, USA) [35].

The structures of six enantiomeric pairs of nAChR ligands T1–T6 (Figure 1, Table 1) were drawn as protonated forms by ChemDraw Ultra 12.0 (PerkinElmer, Waltham, MA, USA). The MM2 force field was used for energy minimization. Then, Gasteiger charges were added to the ligands and the protein templates and saved as a pdbqt file, a supported format of AutoDock4.2, for the molecular docking study.

### 3.2. Molecular docking

The molecular docking of non-flexible amino acid residues was performed by AutoDock4.2 [35], with genetic algorithm (GA) parameters as described by our previous study [45]. The parameters included: 100 GA runs, a population size of 150, a maximum of 10,000,000 evaluations, and a maximum of 27,000 generations. The ligand conformations which have similar 3D orientation within 2.0 Å root-mean-square deviation (RMSD) were grouped as conformation clusters. The docked poses in the highest cluster were analyzed for the number of members in the cluster, free binding energies (ΔGbinding), and ligand efficiency (LE) [46]. The binding interaction between ligand and target protein was visually analyzed by AutoDock4.2, PyMOL (Schrödinger, New York, NY, USA) [47] and MOE (Chemical Computing Group, Montreal, Quebec, Canada) [48]. The cation-π and π-π interactions were visualized by the MOE program.

### 3.3. Molecular Dynamics (MD) Simulation

The resulting complexes were optimized by MD simulation using NAMD software (University of Illinois at Urbana-Champaign, Urbana, IL, USA) [49] with CHARMM force field [50]. The complexes were solvated in the TIP3P model water box. The charge of the system was neutralized with appropriate number of counter ions. Initially, the water box was minimized by conjugate gradient method. Prior to the MD simulation, the system was equilibrated for 200 ps using NPT ensemble at 310 K and 1 atm which was controlled by the Nosé-Hoover Langevin piston method [51] with 2 fs time steps and SHAKE algorithm. Periodic boundary conditions (PBC) and Particle Mesh Ewald (PME) method [51] were used for calculation. The production steps, 50 ns of MD simulations were performed with trajectories saving every 2 ns for analysis. The complexes stability was evaluated using root mean square deviation (RMSD) (Supplemental Figure S1). Finally, the complexes were analyzed by BIOVIA Discovery Studio Visualizer 2017 (Biovia, San Diego, CA, USA).

## 4. Conclusions

The molecular docking and MD simulation studies to reveal features accounting for the subtype selective binding of our previous experimental binding studies suggests that the high selectivity profile of (*S*)-enantiomers of this quinuclidine-triazole series can be attributed primarily to the non-conserved amino acid residues in the complementary subunit comprising the aromatic cage. The key amino acid residue triggering the α3β4 nAChR subtype selectivity is the non-conserved AspB173, whereas the interaction with conserved residues is still necessary for potency. AspB173 can form hydrogen bond and salt bridge interactions with quinuclidine-NH^+^ of the (*S*)-enantiomers leading to a higher selectivity than their (*R*)-enantiomer counterparts. Regarding the α7 subtype, the selectivity profile of the (*R*)-enantiomers is related to interactions with conserved amino acid residues and the homopentameric structure of the α7 nAChR subunit. The present findings strongly suggest that the interaction with specific amino acid residues, particularly with the non-conserved residue in the complementary subunit comprising the aromatic cage of the nAChR binding site, is a key determinant for the nAChR subtype selectivity. As such, the molecular-level understanding derived from *in silico* experiment can provide guidance in the design of new compounds with high selectivity profiles to nAChR subtypes.

## Figures and Tables

**Table 1:** Structures, binding affinities, eudismic ratios, and subtype selectivity ratios of six enantiomeric pairs T1-T6.

	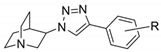 R	K_i_ in nM (Mean ± SD) ^1^		Eudismic Ratio ^2^		Subtype Selectivity Ratio ^3^	
Cpd		α3β4	α7	α3β4 (*S*/*R*)	α7 (*R*/*S*)	α3β4	α7
(*R*)-T1	*p*-F	1010 ± 162	73.0 ± 15		2.4		14
(*S*)-T1		3.09 ± 0.10	174.5 ± 66	327		56	
(*R*)-T2	*m*-F	362 ± 27	117 ± 4		5.7		3.1
(*S*)-T2		2.25 ± 0.42	660.5 ± 139	161		294	
(*R*)-T3	*m*-O(CH_2_)_2_F	558 ± 34	38.8 ± 8		1.9		14
(*S*)-T3		11.8 ± 0.3	74.9 ± 20	47		6.3	
(*R*)-T4	*m*-O(CH_2_)_3_F	1628 ± 11	62.3 ± 10		1.6		26
(*S*)-T4		19.5 ± 0.4	96.9 ± 17	83		5	
(*R*)-T5	*m*-OBn	631 ± 206	22.5 ± 9		12		28
(*S*)-T5		6.67 ± 0.7	279 ± 31	95		42	
(*R*)-T6	*m*-OCH_2_C_6_H_4_-*p*-F	1090 ± 163	33.2 ± 7		4.5		33
(*S*)-T6		7.17 ± 1.2	149 ± 42	152		21	

^1^ Binding affinities are represented by the inhibition constant K_i_. ^2^ Eudismic ratios are reported in terms of the reciprocal values of the K_i_ ratio of (*S*)- to (*R*)-quinuclidine triazole for the α3β4 nAChR and (*R*)- to (*S*)-quinuclidine triazole for α7 nAChR. ^3^ Subtype selectivity ratios are reported in terms of the reciprocal ratio of α3β4 to α7 nAChR K_i_ values for the α3β4 nAChR and α7 to α3β4 nAChR K_i_ values for the α7 nAChR.

**Table 2:** Quality assessment of the selected α7 and α3β4 nAChR homology models.

Selected nAChR Model	Modeller Scoring Function		Ramachandran Plot			
	DOPE Score	GA341 Score	Most Favored Region	Additional Allowed Region	Generously Allowed Region	Disallowed Region
α7	−46,953.29688	1	92.3	6.6	0.5	0.5
α3β4	−46,290.92188	0.99998	91.1	7.6	1.0	0.3

**Table 3:** The amino acid residues involved in the binding interaction to α3β4 and α7 nAChRs.

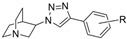		α3β4 nAChR Interaction				α7 nAChR Interaction		
		Hydrogen Bond (Distance in Å)	Salt Bridge Opportunity	Cation-π	π-π	Hydrogen Bond (Distance in Å)	Cation-π	π-π	
Cpd	R								
(*R*)-T1	*p*-F	TyrA93 (2.45) TyrA197 (3.02, 3.16)	-	TrpA149 TyrA197	-	TrpA149 (1.80)	TrpA149 TyrA195	TrpB55
(*S*)-T1		AspB173 (1.78)	AspB173	-	TrpA149	TyrA93 (2.30) SerA148 (3.36)	TyrA188 TyrA195	-
(*R*)-T2	*m*-F	TyrA93 (3.36) TyrA197 (3.23, 3.36)	-	TyrA190 TyrA197	-	TyrA93 (2.24) SerA148 (2.82)	TyrA195	-
(*S*)-T2		AspB173 (1.77)	AspB173	-	TrpA149 TyrA190	TyrA93 (2.11) SerA148 (3.22)	TyrA188 TyrA195	TrpB55
(*R*)-T3	*m*-O(CH_2_)_2_F	AspB173 (2.05) TyrA190 (2.77)	AspB173	-	TyrA190	TrpA149 (1.90) TyrA188 (3.01)	TrpA149 TyrA195	-
(*S*)-T3		AspB173 (1.78)	AspB173	-	TrpA149TyrA190	TrpA149 (1.99)	TrpA149 TyrA195	TrpA149 TrpB55
(*R*)-T4	*m*-O(CH_2_)_3_F	AspB173 (2.08) TyrA190 (2.66)	AspB173	-	TrpA149TyrA190	TyrA93 (2.18) TrpB55 (2.68, 3.12)	-	TrpA149
(*S*)-T4		AspB173 (1.70) TrpB59 (3.39)	AspB173	-	TrpA149	-	TrpB55	TrpA149
(*R*)-T5	*m*-OBn	AspB173 (1.97) TyrA190 (3.19)	AspB173	-	TrpA149	TyrA93 (1.94) TrpB55 (3.01, 3.09)	-	TrpA149
(*S*)-T5		AspB173 (1.65) TrpB59 (3.08, 3.22)	AspB173	-	TrpA149	TyrA93 (1.85) TyrA188 (2.73, 3.37)	-	-
(*R*)-T6	*m*-OCH_2_C_6_H_4_-*p*-F	AspB173 (1.95) TyrA190 (3.15)	AspB173	-	TrpA149 TyrA190	TyrA93 (1.90) TrpB55 (2.83, 3.02)	-	TrpA149
(*S*)-T6		AspB173 (1.66) TrpB59 (3.00, 3.18)	AspB173	-	TrpA149	TyrA93 (1.96) TyrA188 (2.66, 3.32)	-	TyrA188

The hydrogen bonds were analyzed and measured by AutoDock4.2, the cation-π and π-π interactions were analyzed by Molecular Operating Environment (MOE), and the salt bridges were identified through visual inspection. The conserved amino acid residues are represented in bold.

## Supplementary Materials

The following are available online at https://www.mdpi.com/1422-0067/21/17/6189/s1, Figure S1: Root mean square deviation (RMSD) of (a) (*R*)-T2 and (b) (*S*)-T2 with α3β4 nAChR homology model from the initial conformation performed during molecular dynamics simulations, Figure S2: Ramachandran plot assessments of (a) α7 and (b) α3β4 nAChR homology models from PROCHECK, Figure S3: The sequence alignment of AChBP from Lymnaea stagnalis (*Ls*-) and α7, α3 and β4 subtypes of nAChR, Figure S4: The binding interaction of EVP-6124, a reference α7 nAChR ligand to (a) α7 nAChR and (b) α3β4 nAChR, Figure S5: The binding interaction of AT-1001, a reference α3β4 nAChR ligand to (a) α7 nAChR and (b) α3β4 nAChR, Figure S6: The binding interaction of the (*S*)-enantiomer of T1-T6 to α3β4 nAChR, Figure S7: The binding interaction of the (*R*)-enantiomer of T1-T6 to α3β4 nAChR, Figure S8: The binding interaction of the (*R*)-enantiomer of T1-T6 to α7 nAChR, Figure S9: The binding interaction of the (*S*)-enantiomer of T1-T6 to α7 nAChR, Table S1: The binding energy and ligand efficiency of ligands to α3β4 and α7 nAChRs.
