# Supplementary material for: In Silico Finding of Key Interaction Mediated α3β4 and α7 Nicotinic Acetylcholine Receptor Ligand Selectivity of Quinuclidine-Triazole Chemotype
Source: Int J Mol Sci. 2020 Aug 27;21(17):6189. doi: 10.3390/ijms21176189 (PMC7504379; doi:10.3390/ijms21176189)
Supplement: Supplementary file 1 [file ijms-21-06189-s001.pdf]

Supplemental information

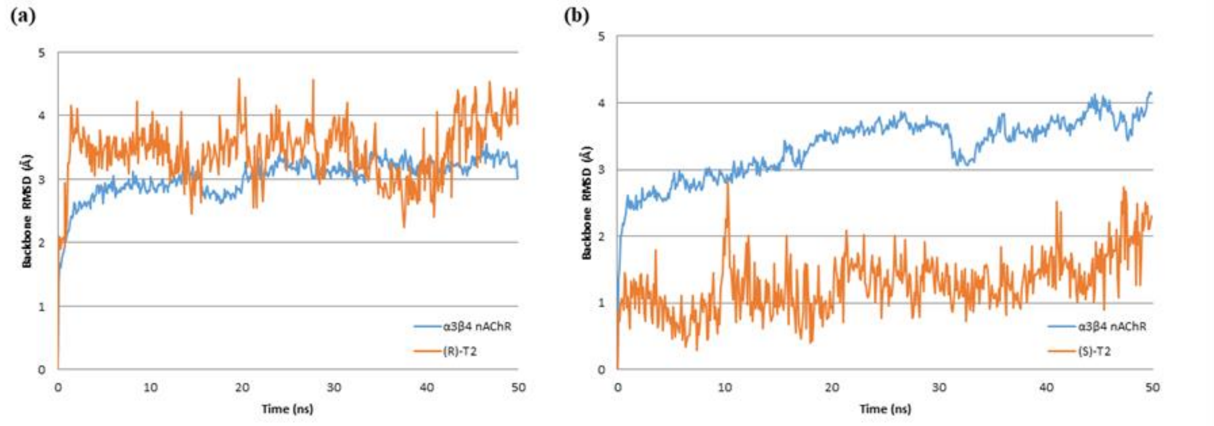

Figure S1. Root mean square deviation (RMSD) of (**a**) (*R*)-T2 and (**b**) (*S*)-T2 with α3β4 nAChR homology model from the initial conformation performed during molecular dynamic simulations.

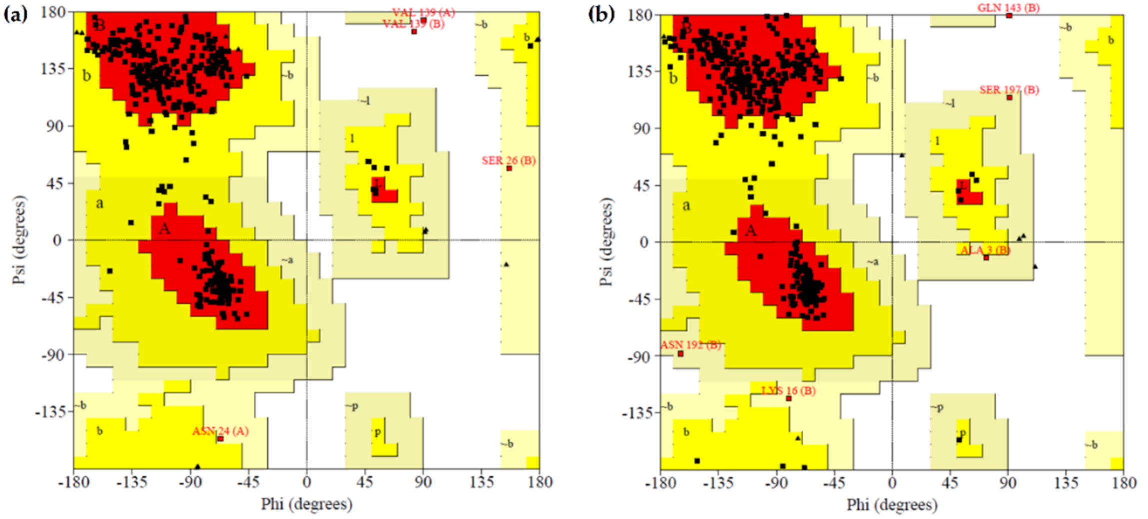

Figure S2. Ramachandran plot assessments of (**a**) α7 and (**b**) α3β4 nAChR homology models from PROCHECK. Red, most favorable region (91.1 and 92.3%); yellow, additional allowed regions (6.6 and 7.6%); light brown, generously allowed region (0.5 and 1.0%); and white, disallowed region (0.5 and 0.3%).

```
Ls-   --LD----RADILYN---IRQTSRPDVIPTQRDRPVAVSVSLKFINILEVNEITNEVDVV    51
α7    --GEFQRKLYKELV--KNYNPLERP---VANDSQPLTVYFSLSLLQIMDVDEKNQVLTTN    53
α3    --SEAEHRLFERLF--EDYNEIIRP---VANVSDPVIIHFEVSMSQLVKVDEVNQIMETN    53
β4    RVANAEEKLMDDLLNKTRYNNLIRP---ATSSSQLISIKLQLSLAQLISVNEREQIMTTN    57

Ls-   FWQQTTWSDRTLAWNSSHSPD--QVSVPISSLWVPDLAAYNAISKP-EVLTPQLARVVSD    108
α7    IWLQMSWTDHYLQWNVSEYPGVKTVRFPDGQIWKPDILLYNSADERFDATFHTNVLVNSS    113
α3    LWLKQIWNDYKLKWNPSDYGGAEFMRVPAQKIWKPDIVLYNNAVGDFQVDDKTKALLKYT    113
β4    VWLKQEWTDYRLTWNSSRYEGVNILRIPAKRIWLPDIVLYNNADGTYEVSVYTNLIVRSN    117

Ls-   GEVLYMPSIRQRFSCDVSGVD-TESGATCRIKIGSWTHHSREISVDPTTENSDDSEYFSQ    167
α7    GHCQYLPPGIFKSSCYIDVRWFPFDVQHCKLKFGSWSYGGWSLDLQMQ--EADISG-YIP    170
α3    GEVTWIPPAIFKSSCKIDVTYFPFDYQNCTMKFGSWSYDKAKIDLVLIGSSMNLKD-YWE    172
β4    GSVLWLPPAIYKSACKIEVKYFPFDQQNCTLKFRSWTYDHTEIDMVLMTPTASMDD-FTP    176

Ls-   YSRFEILDVTQKKNSVTYSCCPEAYEDVEVSLNFRKKGRSEIL    210
α7    NGEWDLVGIPGKRSERFYECCKEPYPDVTFTVTMRRRT-----    208
α3    SGEWAIIKAPGYKHDIKYNCCEEIYPDITYSLYIRRL------    209
β4    SGEWDIVALPGRRTV--NPQDPSYVDVTYDFIIKRKPLFYT-    215
```

Figure S3. The sequence alignment of AChBP from *Lymnaea stagnalis* (*Ls-*) and α7, α3 and β4 subtypes of nAChR. The red box indicated conserved amino acid residues.

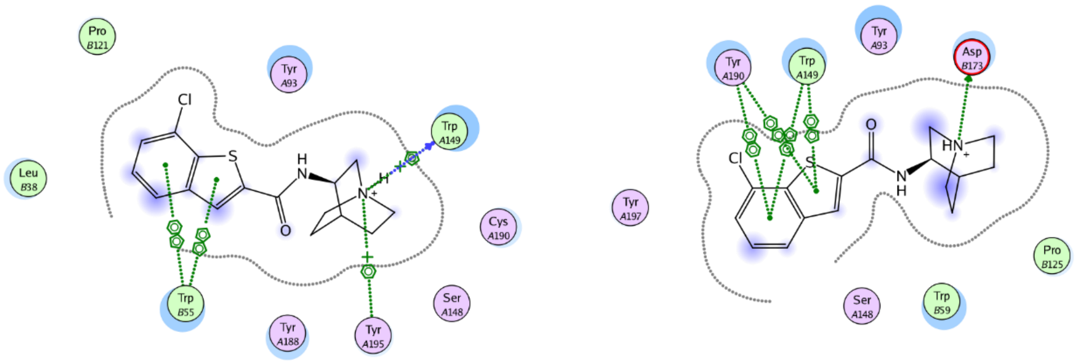

(a) **(b)**

Figure S4. The binding interaction of EVP-6124, a reference α7 nAChR ligand to (**a**) α7 nAChR and (**b**) α3β4 nAChR. The quinuclidine is able to form two cation-π interactions to TrpA149 and TyrA195 and one hydrogen bond interaction with TrpA149 in α7 nAChR, whereas it can interact via hydrogen bond with only AspB173 in α3β4 nAChR. This ligand is capable of forming π-π interaction with the amino acid residues TrpB55 in α7 nAChR and TrpA149 and TyrA190 in α3β4 nAChR. Color schemes for residues and binding interaction: green, hydrophobic residues; light purple, polar residues; red ring, acidic residues; arrow head, hydrogen bond interaction (the donor is at the base of the arrow and the acceptor is at the head); line of benzene ring with plus symbol, cation-π interaction; line of two benzene rings, π-π interaction.

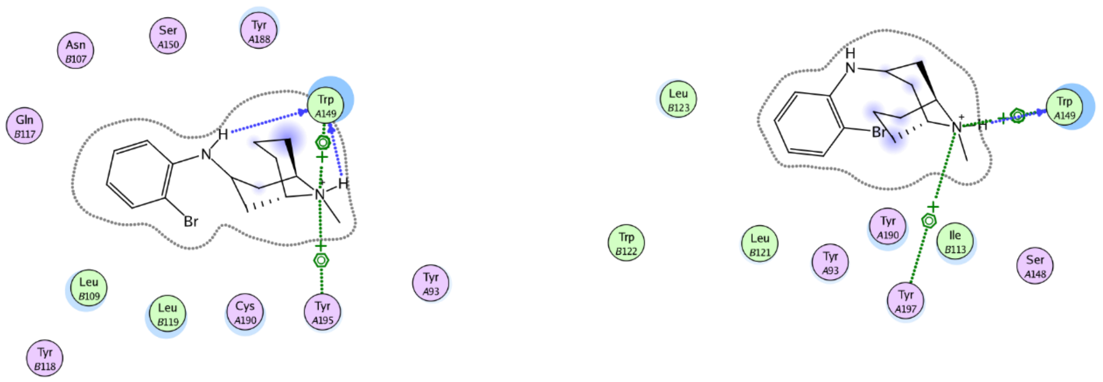

(a) **(b)**

Figure S5. The binding interaction of AT-1001, a reference α3β4 nAChR ligand to (**a**) α7 nAChR and (**b**) α3β4 nAChR. The bicyclic amine is able to form two cation-π interactions to TrpA149 and TyrA195 in α7 nAChR, whereas it can interact with both TrpA149 and TyrA197 via cation-π interaction in α3β4 nAChR. Hence, the hydrogen atom of protonated quinuclidine can form hydrogen bond interaction with the backbone of TrpA149 in both nAChRs and the secondary amine can form hydrogen bond interaction with the backbone of TrpA149 in α7 nAChR. Color schemes for residues and binding interaction: green, hydrophobic residues; light purple, polar residues; arrow head, hydrogen bond interaction (the donor is at the base of the arrow and the acceptor is at the head); line of benzene ring with plus symbol, cation-π interaction.

Table S1. The binding energy and ligand efficiency of ligands to α3β4 and α7 nAChRs.

Compound	α3β4 nAChR			α7 nAChR		
	% Member in the largest cluster	Free binding energy, ΔG	Ligand efficiency (LE)*	% Member in the largest cluster	Free Binding energy, ΔG	Ligand efficiency (LE)*
EVP6124	100	-11.73	-0.56	76	-10.75	-0.51
AT-1001	55	-10.16	-0.56	40	-10.75	-0.6
	31	-9.89	-0.55	25	-9.83	-0.55
(*R*)-T1	63	-11.1	-0.55	91	-10.11	-0.51
(*S*)-T1	100	-10.24	-0.51	91	-9.89	-0.49
(*R*)-T2	66	-11.15	-0.56	70	-11.39	-0.57
(*S*)-T2	100	-10.45	-0.52	42	-9.92	-0.50
				41	-9.88	-0.49
(*R*)-T3	42	-11.43	-0.50	48	-11.54	-0.50
	31	-11.27	-0.49	37	-10.91	-0.47
(*S*)-T3	60	-11.52	-0.50	34	-11.15	-0.48
				32	-10.92	-0.47
(*R*)-T4	51	-11.49	-0.48	38	-11.28	-0.47
	39	-11.94	-0.5	32	-11.75	-0.49
(*S*)-T4	95	-11.85	-0.49	40	-11.44	-0.48
				17	-11.21	-0.47
(*R*)-T5	64	-14.18	-0.53	78	-13.57	-0.50
(*S*)-T5	88	-14.25	-0.53	67	-13.36	-0.49
(*R*)-T6	83	-14.64	-0.52	84	-13.84	-0.49
(*S*)-T6	91	-14.72	-0.53	89	-13.73	-0.49

* LE is the ratio of Gibbs free energy (ΔG) to the number of non-hydrogen atoms of the ligand.

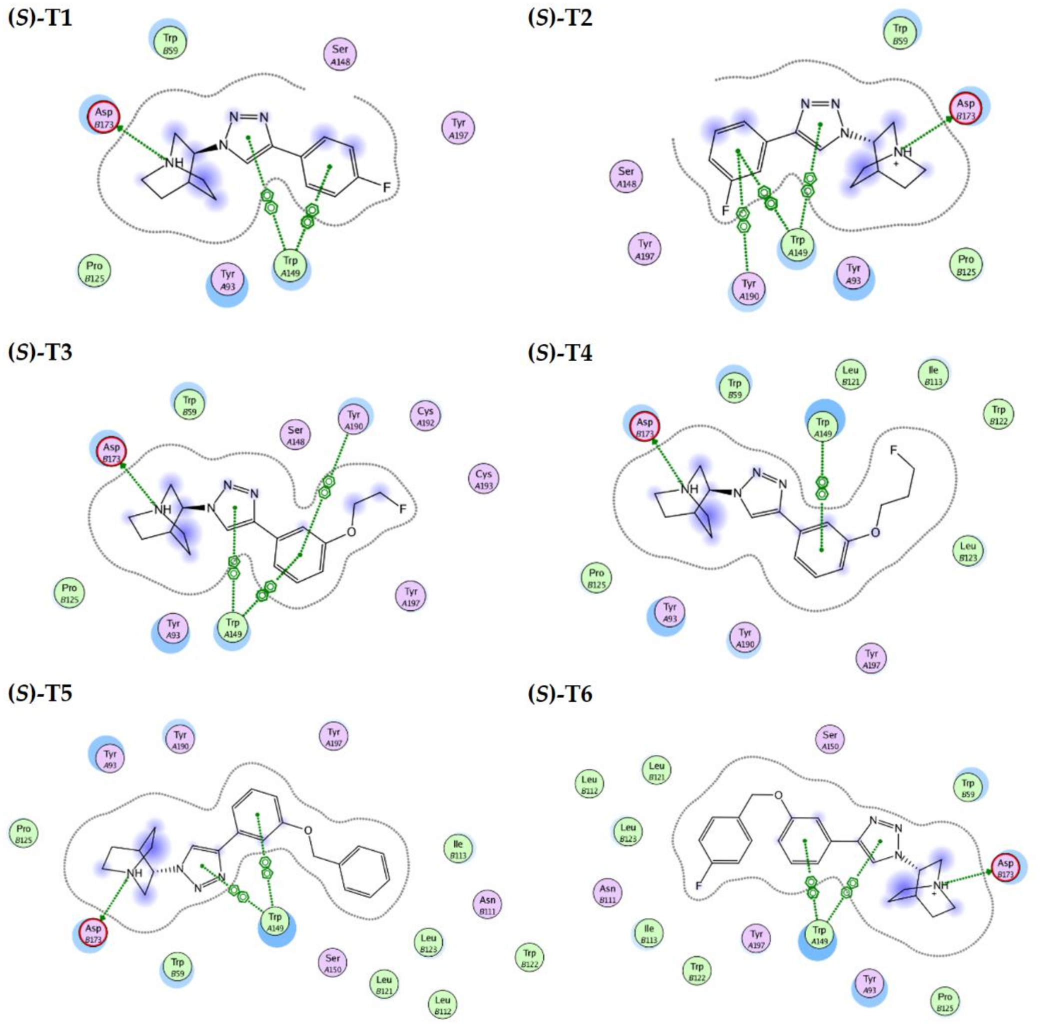

Figure S6. The binding interaction of the (*S*)-enantiomer of T1-T6 to α3β4 nAChR. The green arrow indicated hydrogen bond interaction between the N atom of quinuclidine and AspB173. Color schemes for residues and binding interaction: green, hydrophobic residues; light purple, polar residues; red ring, acidic residues; arrow head, hydrogen-bond interaction (the donor is at the base of the arrow and the acceptor is at the head); line of two benzene rings, π-π interaction.

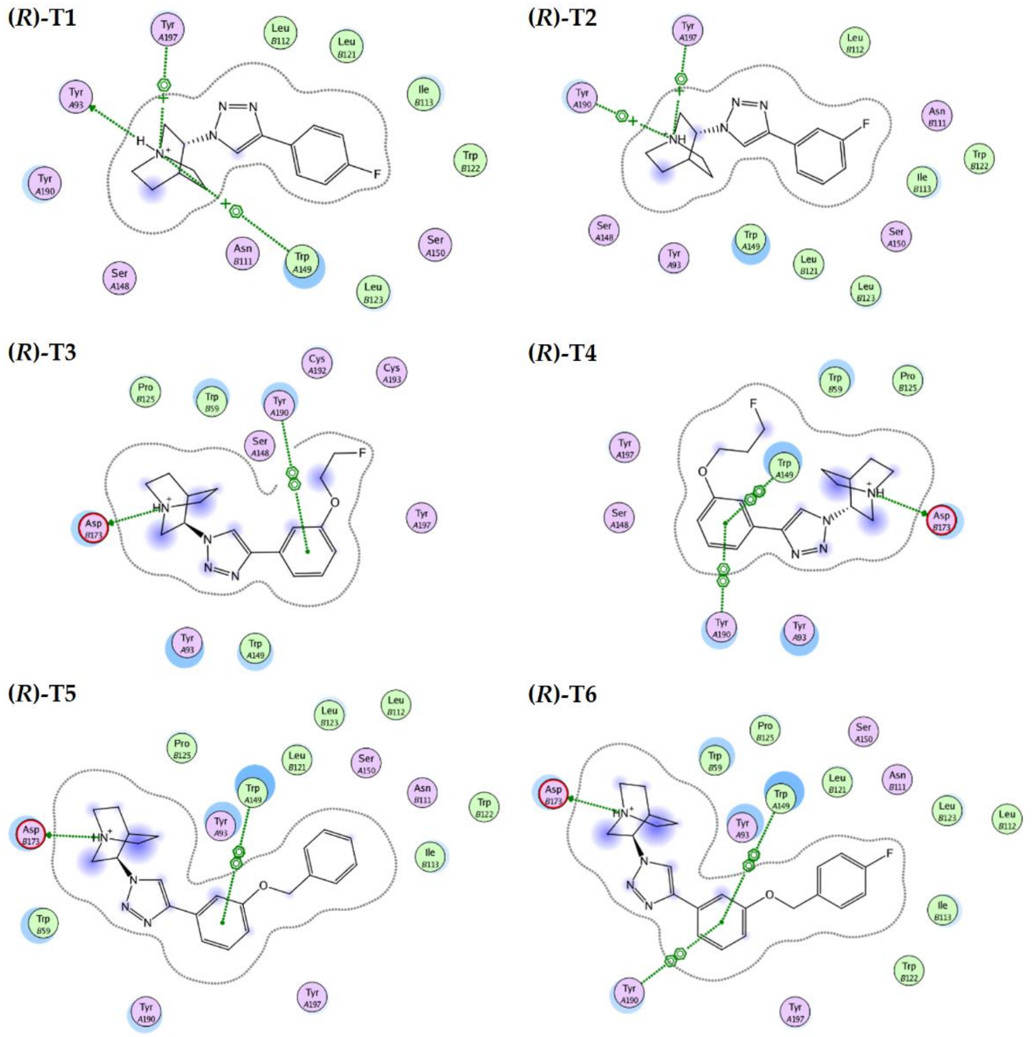

Figure S7. The binding interaction of the (*R*)-enantiomer of T1-T6 to α3β4 nAChR. Color schemes for residues and binding interaction: green, hydrophobic residues; light purple, polar residues; red ring, acidic residues; arrow head, hydrogen bond interaction (the donor is at the base of the arrow and the acceptor is at the head); line of benzene ring with plus symbol, cation-π interaction; line of two benzene rings, π-π interaction.

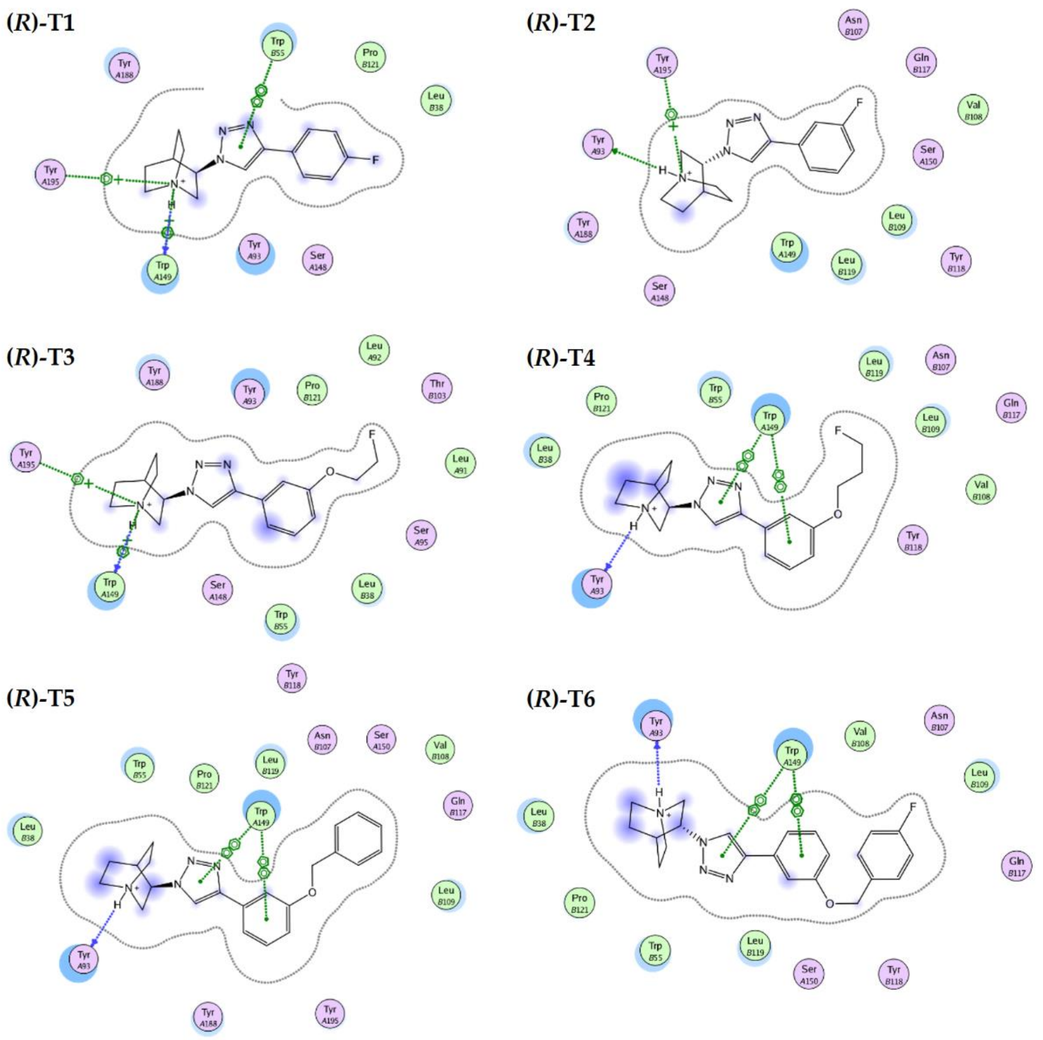

Figure S8. The binding interaction of the (*R*)-enantiomer of T1-T6 to α7 nAChR. Color schemes for residues and binding interaction: green, hydrophobic residues; light purple, polar residues; arrow head, hydrogen bond interaction (the donor is at the base of the arrow and the acceptor is at the head); line of benzene ring with plus symbol, cation-π interaction; line of two benzene rings, π-π interaction.

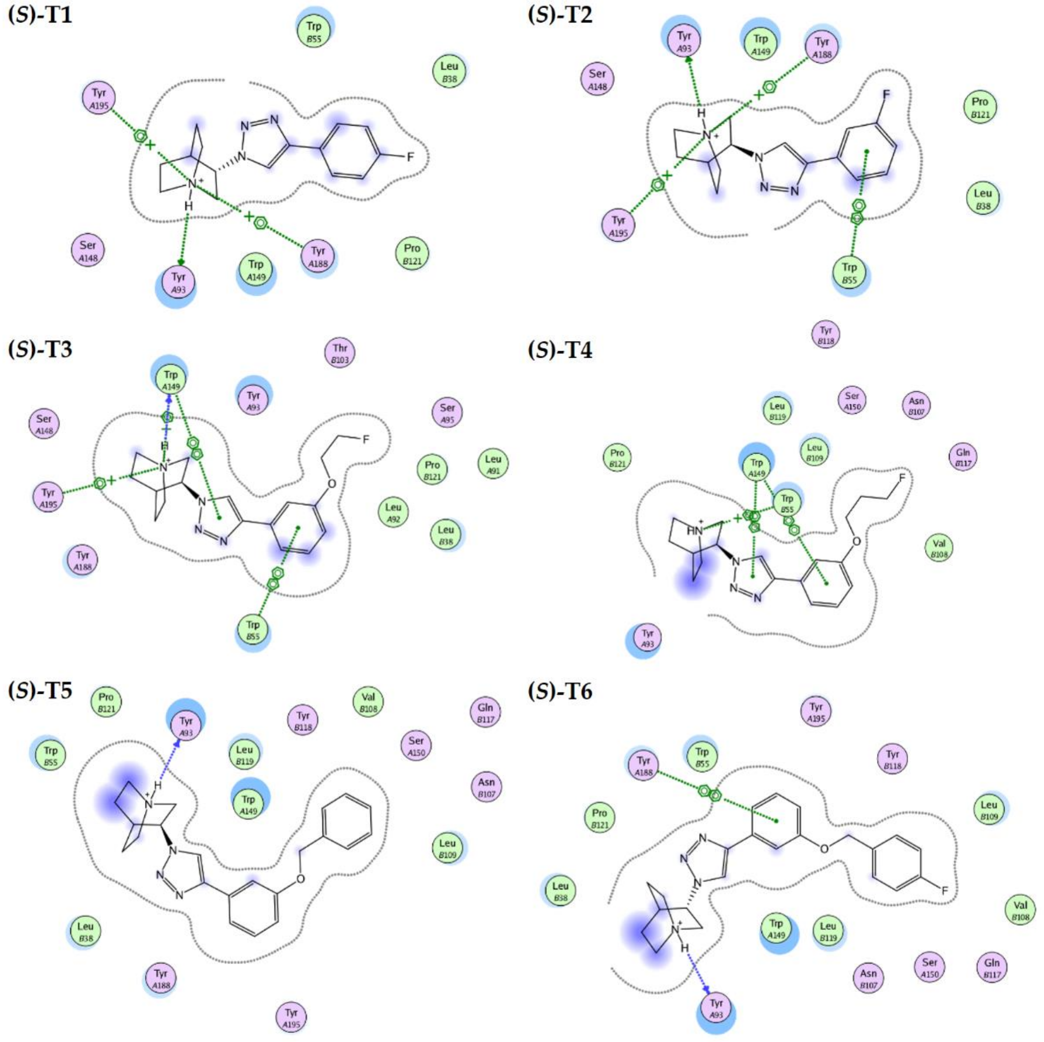

Figure S9. The binding interaction of the (S)-enantiomer of T1-T6 to α7 nAChR. Color schemes for residues and binding interaction: green, hydrophobic residues; light purple, polar residues; line of benzene ring with plus symbol, cation-π interaction; line of two benzene rings, π-π interaction.

.
